# Artificial Intelligence for Integrated Analysis of Non-Blood Biological Fluids: From Biomarker Discovery to Clinical Decision-Support Systems

**DOI:** 10.3390/diagnostics16152478

**Published:** 2026-08-06

**Authors:** Valentina Becherucci, Francesca Romano, Edda Russo

**Affiliations:** 1 Clinical Pathology Laboratory, Santa Maria Annunziata Hospital, Azienda USL Toscana Centro, Bagno a Ripoli, 50012 Florence, Italy; valentina.becherucci@uslcentro.toscana.it; 2 General Laboratory, Azienda Ospedaliero-Universitaria Careggi, 50134 Florence, Italy; 3 Clinical Pathology Laboratory Unit, San. Giuseppe Hospital, Azienda USL Toscana Centro, 50053 Empoli, Italy

**Keywords:** artificial intelligence, clinical decision support systems, non-blood biological fluids, machine learning, multi-omics, precision laboratory medicine

## Abstract

The analysis of non-blood biological fluids, including cerebrospinal fluid (CSF), serous effusions, and synovial fluid, plays a central role in laboratory medicine by providing essential diagnostic and prognostic information for neurological, infectious, inflammatory, and neoplastic diseases. However, the interpretation of these specimens remains challenging because it requires the integration of heterogeneous biochemical, cytological, microbiological, molecular, and clinical data, often in the absence of standardized analytical workflows. Artificial intelligence (AI), particularly Machine Learning (ML) and Deep Learning (DL), is emerging as a powerful approach for extracting clinically relevant information from complex multidimensional datasets beyond the capabilities of conventional analytical methods. AI-driven Clinical Decision-Support Systems (CDSSs) can integrate laboratory findings with clinical, demographic, imaging, and multi-omics data, supporting diagnostic interpretation, patient stratification, and personalized clinical decision-making. At the same time, the convergence of AI with proteomics, metabolomics, metagenomics, and other omics technologies is accelerating biomarker discovery and advancing precision laboratory medicine. Current evidence indicates different levels of maturity across biological fluids. AI-assisted interpretation of CSF biomarkers and digital cytology of serous effusions currently show the strongest clinical evidence, whereas applications involving synovial fluid and integrated multi-omics remain largely exploratory. Although important technical, methodological, and regulatory challenges still limit widespread clinical implementation, AI has the potential to improve diagnostic accuracy, reduce interpretative variability, and support more integrated diagnostic workflows. This mini-review summarizes current and emerging AI applications in non-blood biological fluid analysis, with particular emphasis on biomarker discovery, CDSS, multi-omics integration, current evidence, existing limitations, and future perspectives for precision laboratory medicine.

## 1. Introduction

The analysis of non-blood biological fluids is an essential component of modern laboratory medicine and plays a pivotal role in the diagnosis and monitoring of a broad spectrum of neurological, infectious, inflammatory, and neoplastic diseases. Among these specimens, cerebrospinal fluid (CSF), serous effusions (pleural, peritoneal, and pericardial fluids), and synovial fluid are routinely investigated because they provide biochemical, cytological, microbiological, and molecular information that reflects pathological processes occurring within specific anatomical compartments [1]. In particular, CSF analysis has become indispensable for the diagnostic evaluation of central nervous system disorders [2,3] and is increasingly recognized as a key source of biomarkers for neurodegenerative diseases, including Alzheimer’s disease, where amyloid-β and tau proteins provide valuable insights into the underlying neuropathological processes [2,4].

Despite their considerable clinical value, the diagnostic interpretation of non-blood biological fluids remains challenging throughout the total testing process, encompassing the pre-analytical, analytical, and post-analytical phases. Pre-analytical variables—including specimen collection, limited sample volume, and contamination during sampling—may substantially affect analytical quality. Analytical limitations arise because many laboratory assays have been developed and validated for serum or plasma rather than for alternative biological matrices. Consequently, matrix-specific validation, reference intervals, and analytical standardization remain limited for several non-blood biological fluids. The post-analytical phase is equally demanding, requiring the integration of laboratory findings with clinical presentation, imaging studies, and patient history to achieve an accurate differential diagnosis [1,5].

These challenges have stimulated growing interest in Artificial Intelligence (AI) as a tool capable of supporting the interpretation of complex laboratory data. Rather than replacing conventional laboratory testing, AI—particularly Machine Learning (ML) and Deep Learning (DL)—offers the opportunity to integrate heterogeneous sources of information, identify clinically relevant patterns, and assist diagnostic reasoning in situations where traditional rule-based approaches may be insufficient. This paradigm is especially relevant for non-blood biological fluids, where meaningful interpretation frequently depends on the simultaneous evaluation of biochemical, cytological, microbiological, molecular, and clinical information.

Within this evolving landscape, AI-driven Clinical Decision-Support Systems (CDSSs) represent one of the most promising applications of computational medicine. By integrating laboratory results with demographic characteristics, medical history, imaging findings, and other clinical variables, these systems can generate data-driven diagnostic insights, support patient stratification, and reduce interpretative variability, thereby contributing to more personalized and evidence-based clinical decision-making [4,5].

Against this background, this review provides a comprehensive overview of current and emerging AI applications in the analysis of non-blood biological fluids, with particular emphasis on CDSSs, biomarker discovery, multi-omics integration, current evidence, existing limitations, and future perspectives for precision laboratory medicine.

## 2. Methods

A structured narrative literature review was conducted to identify studies investigating the application of artificial intelligence (AI), machine learning (ML), deep learning (DL), and clinical decision-support systems (CDSSs) in the analysis of non-blood biological fluids. The literature search was performed using PubMed, Scopus, and Web of Science and included articles published between January 2018 and June 2026. The search strategy combined the terms “artificial intelligence”, “machine learning”, “deep learning”, and “clinical decision support systems” with keywords related to “cerebrospinal fluid”, serous effusions, synovial fluid, diagnosis, biomarkers, and laboratory medicine. The strategy was adapted as appropriate for each database. Original articles, systematic reviews, methodological studies, and consensus documents published in English were considered eligible. Following title and abstract screening, potentially relevant publications underwent full-text evaluation. Particular emphasis was placed on studies investigating AI applications in CSF, serous effusions, synovial fluid, and multi-omics integration. The selected studies were subsequently categorized according to biological fluid, AI methodology, and clinical application. Given the narrative nature of this review, no formal meta-analysis was performed. Instead, the discussion focuses on studies reporting diagnostic or prognostic applications of AI, with particular attention to model validation, clinical utility, and translational challenges. To strengthen the critical appraisal of the included studies, a qualitative domain-based assessment was performed using principles derived from PROBAST + AI [6] and TRIPOD + AI [7]. The appraisal considered participant and data-source selection, representativeness of the development cohorts, definition and measurement of predictors and outcomes, sample size, handling of missing data, model-development procedures, potential overfitting, validation strategy, reporting completeness, and applicability to routine clinical practice. Particular attention was given to whether training and test datasets reflected consecutive real-world populations, whether independent external validation was performed, and whether the analytical platforms and clinical settings were sufficiently described to support model reproducibility. Because this was a narrative review and several publications did not provide all information required for a formal domain-level assessment, no overall numerical score or definitive risk-of-bias classification was assigned.

## 3. Diagnostic Complexity of Non-Blood Biological Fluids

The diagnostic evaluation of non-blood biological fluids is inherently more complex than that of conventional blood samples because these matrices are characterized by greater biological heterogeneity and a lower degree of analytical standardization. Unlike serum and plasma, which benefit from well-established analytical protocols and reference intervals, many non-blood biological fluids lack universally accepted procedures and validated diagnostic thresholds, making both analytical reliability and clinical interpretation more challenging [8].

The complexity begins during the pre-analytical phase. Sample collection varies according to the anatomical compartment, while limited specimen volume, inappropriate handling, delayed processing, or contamination may substantially affect analytical performance. Cerebrospinal fluid is particularly vulnerable to blood contamination during lumbar puncture, whereas prolonged storage may compromise cellular integrity and analyte stability, especially in low-volume or low-protein specimens [8,9].

Analytical challenges further complicate interpretation. Many laboratory assays currently used for non-blood biological fluids were originally developed and validated for serum or plasma, and their analytical performance in alternative matrices is often insufficiently characterized. The absence of matrix-specific validation procedures and reference intervals may therefore introduce analytical bias and reduce diagnostic confidence [10].

The post-analytical phase is equally demanding because clinically meaningful interpretation rarely depends on a single laboratory parameter. Instead, it requires the integration of biochemical findings, cytological evaluation, microbiological results, and clinical information. For example, the diagnosis of serous effusions relies on the combined interpretation of protein concentration, lactate dehydrogenase, cellular composition, and clinical presentation, whereas synovial fluid analysis requires the simultaneous evaluation of leukocyte count, crystal identification, inflammatory biomarkers, and patient history to distinguish infectious, inflammatory, and degenerative joint disorders [10].

The multidimensional nature of these diagnostic pathways highlights one of the principal limitations of conventional rule-based interpretation. As laboratory datasets become increasingly heterogeneous, computational approaches capable of integrating multiple sources of information may provide substantial support for diagnostic reasoning. In this context, AI offers the opportunity to move beyond isolated parameter evaluation toward a more comprehensive interpretation of biological complexity [11].

## 4. Artificial Intelligence in Laboratory Medicine

AI is progressively reshaping multiple areas of healthcare, including laboratory medicine, by enabling the extraction of clinically meaningful information from increasingly complex biological datasets. Rather than replacing conventional laboratory diagnostics, AI extends the interpretative capability of laboratory medicine by identifying multidimensional patterns that cannot be readily recognized through traditional statistical or rule-based approaches. ML and DL, the two most widely applied AI methodologies, are particularly suited to this task because they can process high-dimensional data, detect hidden relationships among variables, and generate predictive models from heterogeneous sources of information [12].

The selection of an appropriate AI methodology depends largely on the characteristics of the available biological data and the specific clinical objective. Conventional supervised machine learning approaches, including Random Forest (RF) and Support Vector Machine (SVM), remain widely used in laboratory medicine because they provide robust performance when analysing structured datasets containing biochemical, molecular, or clinical variables [5,11,13]. RF models are particularly advantageous for heterogeneous datasets because they can capture nonlinear interactions among variables, tolerate missing data, and provide measures of feature importance. Conversely, SVM algorithms are especially suitable for high-dimensional datasets with relatively limited sample sizes, a frequent scenario in biomarker discovery studies involving proteomic and metabolomic profiles [4,14,15].

Deep learning approaches have expanded AI applications to domains where raw complex data are available. Convolutional Neural Networks (CNNs) have become the preferred methodology for digital pathology and cytology because they automatically extract morphological features from microscopy images without requiring manual feature engineering [16,17]. More recently, transformer-based architectures have demonstrated potential for multimodal integration by simultaneously analysing laboratory, imaging, and clinical information [18,19]. Graph Neural Networks (GNNs) represent another emerging approach for biological data analysis because they can model complex relationships among genes, proteins, metabolites, and clinical phenotypes within interconnected biological networks [20]. However, these advanced approaches remain at an early stage of clinical implementation in non-blood biological fluid diagnostics and require further validation [21,22,23,24]. Within laboratory medicine, AI has been successfully applied to disease prediction, laboratory test interpretation, workflow optimization, and biomarker discovery. These applications are especially relevant for non-blood biological fluids, where limited sample volume, matrix-specific variability, and the need to integrate biochemical, cytological, microbiological, molecular, and clinical information frequently exceed the capabilities of conventional interpretative strategies [13]. By simultaneously analyzing multiple layers of biological information, AI can support disease classification, identify clinically relevant biomarker signatures, and improve patient stratification across a broad range of neurological, infectious, inflammatory, and neoplastic disorders [14].

DL has further expanded these capabilities through automated image analysis, particularly in digital cytology and digital pathology, where convolutional neural networks can recognize and classify cellular patterns with high diagnostic accuracy. In parallel, AI has accelerated the development of CDSSs, which integrate laboratory findings with demographic, clinical, and imaging data to generate evidence-based diagnostic support and predictive models. Rather than functioning as autonomous diagnostic tools, these systems are designed to augment clinical reasoning, reduce interpretative variability, and facilitate more personalized clinical decision-making [4,11,12,17].

Despite these advances, several barriers continue to limit the routine implementation of AI in laboratory medicine. Data quality, external validation, algorithm transparency, interoperability, and regulatory approval remain major challenges. Consequently, the current role of AI should be viewed as complementary to expert laboratory interpretation rather than as a replacement for professional expertise. Nevertheless, the rapid convergence of AI with digital pathology, multi-omics technologies, and advanced laboratory automation suggests that computational decision support will become an increasingly integral component of future diagnostic workflows, particularly in analytically complex settings such as non-blood biological fluid analysis [21,22,23,24,25].

## 5. AI-Driven Clinical Decision-Support Systems for Biological Fluid Analysis

Within the expanding landscape of AI applications in laboratory medicine, CDSSs represent one of the most clinically relevant developments for the interpretation of non-blood biological fluids. Unlike conventional decision-support approaches based primarily on predefined rules or isolated laboratory parameters, AI-driven CDSSs integrate heterogeneous sources of information—including laboratory findings, patient demographics, clinical history, and imaging data—to generate clinically meaningful diagnostic support.

**Figure 1:**
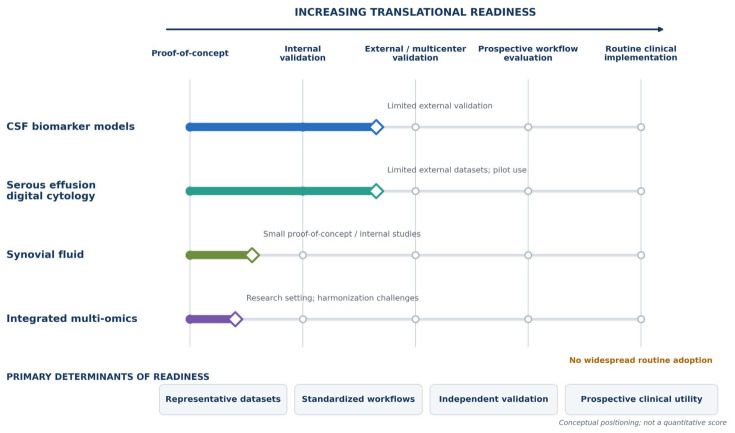
Translational maturity of AI applications in non-blood biological fluid analysis. The continuum ranges from proof-of-concept studies to internal validation, external or multicenter validation, prospective workflow evaluation, and routine clinical implementation. Positions reflect dataset availability and representativeness, analytical standardization, independent validation, and integration into clinical workflows rather than reported diagnostic performance alone. CSF biomarker models and digital cytology of serous effusions currently show the greatest translational maturity, whereas synovial fluid and integrated multi-omics applications remain predominantly at the proof-of-concept or internal-validation stage. None of these applications has yet achieved widespread routine clinical adoption.

By combining ML algorithms with multimodal clinical information, these systems can identify complex biological patterns, improve disease classification, support patient stratification, and reduce interpretative variability [5,11,12,13]. Their greatest value, however, lies not in replacing expert interpretation but in assisting clinicians when diagnostic reasoning requires the simultaneous evaluation of multiple interconnected variables. This capability is particularly relevant for cerebrospinal fluid, serous effusions, and synovial fluid, where clinically meaningful interpretation rarely depends on a single biomarker. Instead, diagnosis typically emerges from the integration of biochemical measurements, cytological findings, microbiological results, molecular analyses, and the overall clinical context. AI-driven CDSSs provide a computational framework capable of integrating these diverse data streams into a unified diagnostic process. Figure 1 illustrates this conceptual workflow. Following appropriate pre-analytical quality assessment and laboratory testing, biochemical, cytological, molecular, omics, imaging, and clinical data are integrated within AI models based on ML and DL methodologies. The resulting computational outputs support biomarker discovery, disease classification, risk stratification, predictive modelling, and ultimately AI-assisted clinical decision-making. The principal AI applications across different biological fluids are summarized in Table 1.

### 5.1. Cerebrospinal Fluid

Among non-blood biological fluids, cerebrospinal fluid (CSF) currently represents the most extensively investigated field for AI applications. The availability of well-characterized biomarkers, relatively standardized analytical workflows, and large neurological datasets has facilitated the development of machine learning (ML) models for disease classification, patient stratification, and biomarker discovery. Nevertheless, most available applications remain research-oriented, and evidence of prospective clinical implementation is still limited.

Published studies have investigated a wide range of data sources, including conventional CSF biomarkers such as amyloid-β, total tau, and phosphorylated tau, as well as proteomic and metabolomic profiles integrated with demographic and clinical information. Most approaches rely on supervised ML methods, including Support Vector Machines, Random Forests, and Artificial Neural Networks, whereas unsupervised methods have been applied to data-driven biomarker profiling and patient stratification.

Bellomo et al. applied an unsupervised Gaussian mixture model to 616 prospectively collected CSF samples from two specialized neurological centres. The use of standardized automated biomarker assays and a two-centre cohort represent a methodological strength. However, the biomarker cut-offs and patient clusters were derived and evaluated within the same study population, and their reproducibility in an independent external cohort was not assessed [14].

Hou et al. developed a Support Vector Machine-based framework using more than 1200 CSF samples obtained from multiple previously published proteomic datasets. The resulting 12-protein panel achieved accuracies exceeding 90% across several datasets and was evaluated in ten cohorts from different countries and across different analytical platforms [26]. This cross-cohort evaluation represents an important strength compared with studies relying exclusively on internal validation. However, the use of secondary datasets resulted in incomplete demographic and clinical information, heterogeneous analytical technologies, and possible overlap between samples included in different datasets. Moreover, validation against other neurodegenerative diseases was limited, and prospective longitudinal validation in routine clinical populations was not performed. Therefore, the reported performance cannot yet be considered evidence of clinical effectiveness.

Tiwari et al. analysed CSF biomarker data from the National Alzheimer’s Coordinating Center database and reported an accuracy of 84.4% for the binary classification of cognitively normal individuals versus patients with dementia stages using Clinical Dementia Rating-based groups [27]. However, the study relied on internal cross-validation, excluded records with missing biomarker or cognitive data, and included markedly imbalanced diagnostic subgroups. In particular, the severe-dementia group was relatively small. No independent external validation or prospective clinical evaluation was performed, and the study was published as a preprint. These limitations increase the possibility of optimistic performance estimates and restrict the generalizability of the model.

Beyond neurodegenerative diseases, CSF molecular, proteomic, and metagenomic studies in central nervous system infections and neuro-oncology generate high-dimensional datasets that may be suitable for AI-assisted analysis. However, much of the current literature establishes the feasibility of biomarker or sequencing-based approaches rather than validating complete AI-driven prediction models or clinical decision-support systems [1,31,32]. These applications should therefore be regarded as exploratory.

Overall, CSF represents the non-blood biological fluid with the most developed data infrastructure and the largest number of AI-related studies. However, the methodological robustness of the evidence remains variable. High reported accuracies are frequently derived from retrospective or selected datasets, and independent prospective validation, calibration assessment, evaluation of clinical utility, and integration into routine laboratory workflows remain uncommon. Current AI models should therefore be considered investigational decision-support tools rather than clinically validated alternatives to established diagnostic pathways.

### 5.2. Serous Effusions

Serous effusions represent one of the most promising areas for AI-assisted digital pathology because diagnostic interpretation frequently depends on the recognition of subtle cytological features together with biochemical and clinical information. Recent advances in digital cytology and DL have substantially improved the automated detection and classification of malignant cells, opening new opportunities for AI-assisted cytopathology. The application of AI to the analysis of serous effusions has primarily focused on improving the etiological classification of pleural, peritoneal, and pericardial fluids, particularly by distinguishing benign from malignant effusions and supporting the diagnosis of infectious and inflammatory diseases. This represents a challenging diagnostic scenario because the interpretation of serous fluids often requires the integration of biochemical, cytological, and clinical information. To address this complexity, published studies have explored a variety of data sources, including biochemical parameters such as protein concentration, lactate dehydrogenase, pH, and glucose levels, cytological findings and cellular composition, digital whole-slide cytology images, as well as demographic and clinical variables. Most AI applications in this field rely on supervised classification algorithms or automated image analysis approaches based on deep learning techniques. Additional methodologies include object detection for cellular identification, image segmentation, and predictive models designed to improve diagnostic accuracy and workflow efficiency. Recent evidence has been particularly encouraging in the area of digital cytopathology [16,28,29]. Park et al. developed a deep convolutional neural network for pleural fluid cytology that reported an accuracy of 81.1%, a sensitivity of 95.0%, and a specificity of 98.6% for malignant-cell detection, providing valuable support to conventional cytological evaluation [16]. Similarly, Kim et al. proposed a whole-slide image-based deep learning model capable of discriminating malignant from benign pleural effusions, reporting an area under the curve (AUC) of 0.97 and a diagnostic accuracy approaching 97% [28]. More recently, Giarnieri et al. introduced user-friendly AI-assisted image analysis systems based on object-detection algorithms, including YOLO architectures, demonstrating the potential of these tools to simplify cytological interpretation while maintaining high diagnostic performance [29]. Despite these promising results, several limitations should be acknowledged. Most available studies are retrospective and rely on highly selected image datasets rather than consecutive routine clinical samples. In addition, algorithm development still depends heavily on manual image annotation by expert cytopathologists, and external multicenter validation remains limited. Consequently, although AI-assisted cytology shows considerable promise for the evaluation of serous effusions, further prospective studies based on standardized digital pathology workflows are needed before widespread implementation in routine laboratory practice can be recommended.

### 5.3. Synovial Fluid

Compared with cerebrospinal fluid and serous effusions, artificial intelligence (AI) applications in synovial fluid analysis remain at an earlier stage of development. Nevertheless, the integration of machine learning (ML) with routinely generated laboratory data, digital microscopy, and spectroscopic technologies has potential to support the differential diagnosis of septic arthritis, inflammatory arthropathies, crystal-induced arthritis, and degenerative joint diseases. Synovial fluid interpretation is intrinsically complex because it requires the combined assessment of total and differential cell counts, crystal morphology, inflammatory biomarkers, microbiological findings, and relevant clinical information. AI-based approaches may facilitate the integration of these heterogeneous data and identify diagnostically relevant patterns that may not be apparent when individual variables are considered separately.

Current studies have investigated leukocyte counts and differentials, crystal identification, Raman spectra, inflammatory biomarkers, and demographic and clinical variables. Most published models use supervised ML for classification and predictive modelling, with particular emphasis on spectroscopic data. In the most representative study, Niessink et al. applied principal component analysis followed by support vector machine classification to Raman spectra obtained from synovial fluid samples from 446 patients. The model achieved an overall accuracy of 88.0% for the classification of pathological crystals; the reported accuracies were 92.5% for monosodium urate and 96.0% for calcium pyrophosphate when evaluated against the corresponding ACR/EULAR classification criteria [30]. These results demonstrate the technical feasibility of automated spectral interpretation, but they were obtained using a specialised Raman spectroscopy platform and should not be interpreted as evidence of immediate applicability across routine clinical laboratories.

From an implementation perspective, a clear distinction should therefore be made between AI applications based on routinely available laboratory inputs and those requiring advanced analytical technologies. Models using total and differential cell counts, inflammatory biomarkers, clinical variables, or digital images acquired through conventional or compensated polarised-light microscopy would be more compatible with existing laboratory workflows and would require comparatively limited additional infrastructure. Such approaches could potentially support the differentiation of septic, inflammatory, crystal-induced, and degenerative joint diseases or improve the reproducibility of crystal identification. Nevertheless, technological compatibility with routine practice should not be considered equivalent to clinical readiness. Evidence specifically validating AI models based on these routine inputs remains limited, and their clinical utility has not yet been established through independent external validation or prospective multicentre studies.

By contrast, Raman spectroscopy and other advanced spectroscopic or multi-omics platforms require dedicated instrumentation, standardised acquisition and preprocessing procedures, specialised quality-control systems, and technical expertise that are not routinely available in most clinical laboratories. Their reproducibility may be influenced by differences in instrumentation, sample preparation, acquisition protocols, and spectral preprocessing. The high diagnostic performance reported in experimental studies should therefore be interpreted within the context of the analytical platform, cohort selection, and validation strategy. Near-term translational research should prioritise externally validated models based on routinely generated laboratory and microscopic data, whereas Raman spectroscopy-based and multi-omics applications should currently be regarded as research-oriented or proof-of-concept approaches.

More broadly, differences in translational maturity across non-blood biological fluids appear to reflect the structure and robustness of the available evidence rather than the intrinsic suitability of a particular fluid for AI analysis. CSF applications benefit from well-characterised biomarkers, comparatively standardised analytical workflows, established neurological cohorts, and larger datasets [14,26,27]. However, their clinical readiness remains constrained by the predominance of retrospective designs and insufficient prospective and external validation. AI-assisted cytology of serous effusions benefits from the digital nature of image-based data and advances in computer vision [16,28,29]. Nevertheless, many models have been developed using curated image datasets, expert-selected regions, or manual annotations, with limited evaluation in consecutive real-world samples. Synovial fluid applications remain less mature because available datasets are generally smaller and less standardised, the target diseases are heterogeneous, and the most developed models depend on specialised technologies that are not widely accessible [30]. Integrated multi-omics approaches face additional challenges related to high dimensionality relative to cohort size, missing data, batch effects, cross-platform harmonisation, interpretability, and cost, as discussed in Section 6.

Thus, the number of published studies alone does not explain the different maturity levels. Dataset size and representativeness, pre-analytical and analytical standardisation, technological accessibility, independent external validation, and integration into prospective clinical workflows are the principal determinants of translational readiness. As summarised in Figure 1, CSF biomarker models and digital cytology of serous effusions currently occupy comparatively more advanced positions along the translational pathway, whereas synovial fluid and integrated multi-omics applications remain predominantly at the proof-of-concept or internal-validation stage. Figure 1 provides the overall comparison of translational maturity, whereas Table 1 presents a structured, study-level critical appraisal of representative investigations. Importantly, none of these applications has yet achieved widespread routine clinical implementation. Current AI systems should therefore be regarded as tools that support, rather than replace, expert laboratory and clinical interpretation.

## 6. Integration of Multi-Omics and AI in Biological Fluid Diagnostics

The convergence of multi-omics technologies and artificial intelligence is expanding the diagnostic potential of non-blood biological fluids. High-throughput platforms, including proteomics, metabolomics, and metagenomics, can characterise molecular signatures associated with neurological, infectious, inflammatory, and neoplastic diseases. Unlike single-analyte approaches, these technologies capture complementary biological layers and may provide a more comprehensive representation of disease processes. Their high dimensionality, however, also creates substantial analytical challenges related to limited sample size, missing data, batch effects, correlated variables, and cross-platform harmonisation.

Computational biology has consequently shifted from single-layer biomarker discovery towards systems-level integration. Network biology represents molecular alterations as interconnected pathways, regulatory modules, and disease-associated hubs, whereas Bayesian approaches can combine heterogeneous data while explicitly modelling uncertainty, prior knowledge, and incomplete observations [33,34]. Graph-based learning, including graph neural networks, may further represent relationships among genes, proteins, metabolites, microorganisms, and clinical variables [20,34]. These methods are promising for biomarker prioritisation and disease classification but remain largely experimental in laboratory medicine. Machine learning and deep learning should therefore be viewed as analytical tools for integrating complex data rather than as evidence, by themselves, of clinical readiness.

Among the individual omics disciplines, proteomics currently has the most developed evidence base in cerebrospinal fluid particularly for neurodegenerative disorders. Hou et al. integrated proteomic data from more than 1200 CSF samples and used a support vector machine-recursive feature elimination strategy to identify a 12-protein panel that achieved an overall diagnostic accuracy exceeding 90% across multiple Alzheimer’s disease datasets [26]. Nevertheless, some datasets included overlapping patient populations, performance varied across analytical platforms, and longitudinal validation is still required. Bader et al. developed a reproducible mass-spectrometry workflow and analysed more than 1000 proteins in 197 individuals from three studies, identifying CSF proteomic alterations associated with Alzheimer’s disease and a glycolytic signature with potential clinical relevance [35]. More recently, Scalia et al. combined data-independent acquisition proteomics with ML in 138 individuals and identified a 15-protein classifier capable of distinguishing two Alzheimer’s disease subtypes and non-Alzheimer controls, with additional evaluation in publicly available datasets [36]. These studies support the potential of AI-assisted CSF proteomics, but they do not yet establish a standardised or prospectively validated clinical pathway.

Metabolomics provides complementary information by capturing dynamic alterations in lipid, energy, amino acid, and oxidative-stress pathways. ML may be useful in this setting because metabolomic datasets contain numerous highly correlated variables. In a quantitative nuclear magnetic resonance study, Berezhnoy et al. identified CSF and serum metabolic changes associated with mild cognitive impairment and Alzheimer’s disease, including alterations in acetoacetate, valine, and other energy-related metabolites [37]. However, the cohort was modest, sex distributions were imbalanced in some groups, and the study mainly demonstrated metabolomic differentiation rather than a clinically validated AI diagnostic model. Evidence for metabolomic applications in pleural and synovial fluids is even more limited and should currently be regarded as exploratory.

Metagenomics has broadened the diagnostic scope of biological-fluid analysis, particularly for central nervous system infections. Metagenomic next-generation sequencing (mNGS) enables untargeted detection of bacterial, viral, fungal, and parasitic nucleic acids directly from clinical specimens. In a prospective multicentre study of patients with meningitis or encephalitis, CSF mNGS provided additional diagnoses beyond conventional testing while also revealing practical limitations related to host background, contamination, turnaround time, and interpretation [31]. Computational pipelines support sequence classification, pathogen prioritisation, and contamination filtering; AI and ML may further assist these tasks, but their contribution should be distinguished from the broader bioinformatic workflow [32]. Standardized pipelines, quality-control procedures, curated reference databases, and harmonised interpretation criteria remain necessary before widespread routine implementation.

### Disease-Oriented Integration: Alzheimer’s Disease as a Model

**Figure 2:**
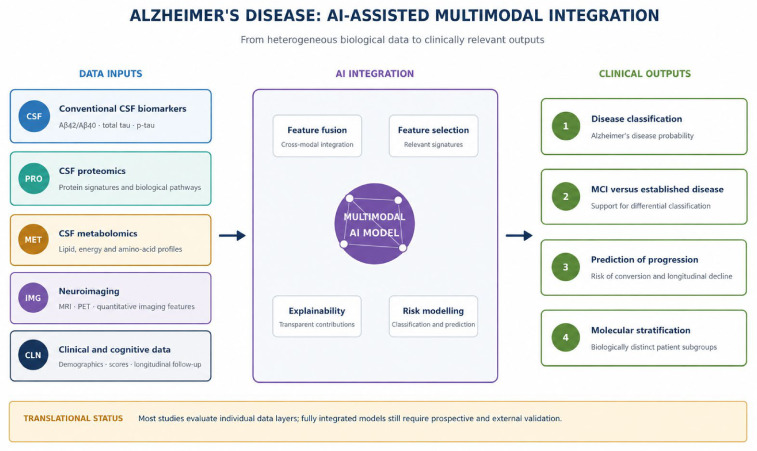
Disease-oriented framework for AI-assisted multimodal integration in Alzheimer’s disease. Conventional CSF biomarkers, proteomic and metabolomic signatures, neuroimaging findings, and clinical and cognitive information can be integrated through AI models to support disease classification, differentiation of mild cognitive impairment from established Alzheimer’s disease, prediction of disease progression, and molecular patient stratification. The figure represents a translational framework because most published studies have evaluated individual data layers or limited combinations, whereas fully integrated models still require prospective and external validation. The arrows illustrate the conceptual workflow from multimodal biological data acquisition through AI-based integration to clinically relevant outputs.

Alzheimer’s disease provides a clinically relevant example of how AI could connect multi-omics data with the diagnostic questions discussed in the previous sections. Current assessment already combines core CSF biomarkers, including amyloid-β42 or the amyloid-β42/40 ratio, total tau, and phosphorylated tau, with cognitive evaluation and neuroimaging. Proteomics may extend this framework by identifying disease-associated protein signatures, whereas metabolomics may capture complementary alterations in lipid, energy, and amino acid metabolism. In principle, AI models could integrate these data with demographic, clinical, imaging, and longitudinal variables to support disease classification, distinguish mild cognitive impairment from established Alzheimer’s disease, estimate progression risk, and identify biologically distinct patient subgroups. Figure 2 summarises this proposed pathway from standardised CSF processing and multi-omics data generation to AI-assisted clinical interpretation.

The available studies support individual components of this framework rather than the fully integrated pathway. Hou et al., Bader et al., and Scalia et al. demonstrated the diagnostic or stratification potential of CSF proteomics, whereas Berezhnoy et al. identified complementary metabolomic differences [26,35,36,37]. These layers have generally been analysed separately. Models that simultaneously combine conventional CSF biomarkers, proteomics, metabolomics, imaging, and longitudinal clinical outcomes remain uncommon and lack sufficient prospective external validation. Accordingly, the Alzheimer’s disease example should be interpreted as a disease-oriented translational framework illustrating potential added value, not as an established AI-based diagnostic pathway.

The same conceptual approach may be extended to other fluids and clinical questions. In malignant serous effusions, for example, AI-assisted digital cytology could potentially be combined with proteomic, metabolomic, cell-free nucleic-acid, and clinical data to improve malignant-cell detection, tumour classification, and treatment monitoring. Current evidence, however, predominantly concerns image-based cytology models evaluated separately from molecular data [16,28,29]. Fully integrated multimodal models for serous effusions therefore remain at the proof-of-concept stage. This contrast illustrates that translational maturity depends not only on algorithmic capability, but also on standardised specimen processing, validated biomarkers, representative datasets, and cohorts linking molecular profiles to clinically meaningful outcomes.

A critical appraisal of the multi-omics literature identifies recurring risks of bias. Many studies are retrospective, single-centre, or based on small and highly selected cohorts; preprocessing and feature-selection procedures are heterogeneous; and high dimensionality relative to sample size increases the risk of overfitting and data leakage. Internal cross-validation is common, whereas independent external validation, calibration assessment, prospective evaluation, and analysis of clinical utility remain limited. Training data may also underrepresent real-world variation in age, comorbidity, disease stage, sample quality, and analytical platform. Study interpretation should therefore consider risk of bias and applicability using frameworks such as PROBAST + AI, together with transparent reporting according to TRIPOD + AI [6,7].

Explainable artificial intelligence (XAI) is also relevant to the clinical translation of multi-omics models. Feature-attribution methods such as SHapley Additive exPlanations (SHAP) and Local Interpretable Model-Agnostic Explanations (LIME) can identify variables contributing to a prediction and may assist biological interpretation [38,39]. Their outputs do not, however, prove biological causality and may be unstable when features are strongly correlated, as frequently occurs in omics datasets. Causal-inference approaches may help distinguish predictive association from plausible biological mechanisms, but they require explicit assumptions and appropriate study designs [40].

Overall, the principal potential advantage of AI lies in connecting complementary data layers to clinically defined diagnostic and prognostic questions rather than analysing each omics dataset in isolation. Current evidence is strongest for selected single-omics applications, particularly AI-assisted CSF proteomics, and substantially weaker for fully integrated multi-omics models. Molecular and multimodal applications in serous effusions and synovial fluid are less mature. Integrated multi-omics should therefore be regarded as a promising translational framework that still requires harmonised pre-analytical and analytical workflows, representative multicentre datasets, independent external validation, interoperability with laboratory information systems, and prospective demonstration of clinical utility and cost-effectiveness before routine implementation.

## 7. Challenges and Limitations

Despite the growing interest in artificial intelligence (AI)-assisted analysis of non-blood biological fluids, several scientific, technical, organisational, regulatory, and ethical challenges continue to limit widespread clinical implementation. One of the principal obstacles concerns the availability, quality, and representativeness of the datasets used for model development. Most published studies remain retrospective, include relatively small or highly selected patient cohorts, and rely predominantly on internal validation. Consequently, reported performance may not be reproducible across institutions, analytical platforms, patient populations, or routine clinical settings [21,41].

### 7.1. Laboratory-Specific Implementation Barriers

The implementation of AI in non-blood biological fluid diagnostics is particularly sensitive to pre-analytical variability because these specimens do not represent a single homogeneous analytical matrix. Cerebrospinal fluid, pleural, peritoneal, pericardial, and synovial fluids differ substantially in composition, cellularity, viscosity, protein concentration, and susceptibility to degradation or contamination. Collection container, anatomical sampling site, anticoagulant use, available volume, transport time and temperature, centrifugation, storage conditions, freeze–thaw cycles, blood contamination, and delayed cellular processing may all modify the variables used as model inputs. Unless these factors are standardised and recorded as structured metadata, AI models may learn site-specific pre-analytical artefacts rather than reproducible biological patterns. Laboratories should therefore define fluid-specific standard operating procedures, sample acceptance and rejection criteria, processing timelines, and minimum metadata requirements before model development or clinical implementation [21].

Matrix-specific analytical validation represents a second critical requirement. Many assays used for non-blood biological fluids were originally designed and validated for serum or plasma, and their analytical performance cannot automatically be transferred to alternative matrices. Before laboratory data are used as AI inputs, precision, linearity, limits of detection and quantification, interference, stability, carryover, and matrix effects should be verified for the relevant fluid and analytical platform. The limited availability of matrix-specific reference intervals or validated clinical decision limits further complicates model training and interpretation. Because reference samples from healthy individuals are often difficult or ethically impossible to obtain for several fluids, clinically validated decision limits and context-specific interpretative criteria may sometimes be more appropriate than conventional population-based reference intervals. Models trained using locally defined thresholds may otherwise generate apparently accurate predictions that cannot be transferred to laboratories using different methods or interpretative criteria.

Variability also extends to analytical platforms, calibration procedures, reagent lots, feature-extraction strategies, and computational pipelines. Such heterogeneity reduces data comparability across institutions and limits the transferability of AI models developed from single-centre datasets. These sources of variation should be documented and incorporated into validation and change-control procedures rather than treated as incidental technical details [21].

Integration into existing laboratory digital infrastructure represents another frequently underestimated barrier. Most published AI models are developed and tested offline, whereas routine implementation requires reliable bidirectional communication among analytical instruments, middleware, Laboratory Information Systems (LISs), Electronic Health Records (EHRs), digital cytology platforms, and clinical decision-support interfaces. This integration requires standardised representation of specimen type and anatomical source, analytical method, measurement units, instrument flags, image data, missing values, pre-analytical conditions, and relevant clinical information. Legacy LIS architectures, proprietary instrument formats, incomplete metadata, and inconsistent coding systems may prevent automated data exchange or introduce transcription and mapping errors. Operational deployment should therefore include model-version traceability, audit logs, role-based access, human override mechanisms, downtime procedures, and clear rules governing how AI-generated outputs are displayed, verified, authorised, and released [41].

AI models should be managed as continuously monitored components of the total testing process rather than as static software products. Before implementation, laboratories should perform local verification using samples representative of their patient population, analytical platforms, and routine workflow. A prospective silent-deployment phase, in which AI outputs are compared with routine expert interpretation without influencing patient reports, may help identify unexpected errors and workflow-related problems. Post-deployment quality assurance should include monitoring of missing or out-of-distribution inputs, calibration drift, subgroup performance, false-positive and false-negative rates, user overrides, turnaround time, and workload effects. Revalidation should be considered after changes in collection procedures, analytical platforms, reagent lots, software versions, patient populations, or laboratory workflows. Responsibilities for investigating discordance between AI output and expert interpretation must also be explicitly assigned within the laboratory governance structure [41,42].

### 7.2. Regulatory, Ethical, and Governance Considerations

Regulatory, ethical, and governance requirements extend beyond obtaining initial approval for an AI model. In the European Union, the regulatory qualification of AI-based laboratory software depends on its intended medical purpose and may involve the Medical Device Regulation or the In Vitro Diagnostic Medical Device Regulation, together with requirements applicable to high-risk systems under the European Artificial Intelligence Act. Regulatory assessment should therefore consider not only diagnostic accuracy but also analytical and clinical validity, robustness, reproducibility, cybersecurity, human oversight, change-control procedures, and continuous performance monitoring. These considerations are particularly important for adaptive or periodically updated models, whose performance may change after deployment [43,44]. In the United States, regulatory pathways for AI-enabled medical devices developed by the U.S. Food and Drug Administration similarly emphasise safety and effectiveness, appropriate evidence, transparency, and performance management throughout the product lifecycle [45,46].

Ethical governance should address data minimisation, secondary use of clinical data, patient privacy, cybersecurity, and bias arising from underrepresented patient populations. Model interpretability represents an additional concern: the limited transparency of complex deep learning architectures may hinder clinical acceptance and reduce confidence among laboratory professionals and clinicians, who ultimately remain responsible for diagnostic decision-making.

Explainability should therefore be clinically meaningful and should allow laboratory professionals to understand which analytical or clinical variables contributed to a prediction, particularly when an AI output conflicts with established diagnostic criteria or expert interpretation [38,39]. Clear accountability is also required to define responsibilities among the laboratory professional, healthcare institution, software developer, and manufacturer when an AI-assisted recommendation contributes to an erroneous or delayed result. Human oversight remains essential to reduce automation bias and ensure that AI-generated outputs are interpreted within the complete analytical and clinical context [47].

Another critical limitation is the discrepancy between retrospective performance and prospective clinical effectiveness. Although many AI models demonstrate high diagnostic accuracy in retrospective datasets, performance may decrease during external validation because of dataset shift, population differences, analytical variability, and limited interoperability among healthcare systems. Prospective multicentre implementation studies are therefore needed to evaluate not only diagnostic accuracy but also turnaround time, laboratory efficiency, clinical decision-making, cost-effectiveness, patient outcomes, and the frequency with which AI recommendations are accepted or overridden.

Ultimately, successful implementation will require harmonised pre-analytical and analytical procedures, comprehensive metadata documentation, robust external validation, transparent model development, and continuous performance monitoring after deployment. Close collaboration among laboratory professionals, clinicians, information-technology specialists, data scientists, healthcare institutions, manufacturers, and regulatory authorities will be essential to ensure that AI-assisted tools are analytically reliable, clinically useful, operationally sustainable, and safe for patients. In this setting, AI should support rather than replace expert laboratory and clinical interpretation [21,41].

## 8. Future Perspectives

The future adoption of artificial intelligence-driven tools in biological fluid analysis will likely depend less on incremental gains in algorithmic performance than on the establishment of robust analytical and digital infrastructures capable of supporting safe, reproducible, and effective implementation in routine clinical practice. Advances in computational resources, data harmonisation frameworks, and Clinical Decision-Support Systems are enabling increasingly sophisticated models that integrate laboratory, clinical, imaging, and genomic information within unified decision-making frameworks [48,49].

Achieving this transition will require high-quality, well-curated, and representative datasets; standardised pre-analytical and analytical workflows; robust external validation; and seamless interoperability with Laboratory Information Systems and Electronic Health Records. Future implementation will also require careful evaluation of economic sustainability, reimbursement strategies, workflow integration, and professional acceptance. AI systems that cannot be incorporated efficiently into existing diagnostic pathways, LIS, and EHR are unlikely to achieve widespread adoption, even when they demonstrate high performance in retrospective studies. Accordingly, implementation studies should evaluate not only diagnostic accuracy but also turnaround time, laboratory efficiency, clinical decision-making, cost-effectiveness, and patient outcomes, thereby providing evidence of the overall clinical and organisational value of AI-assisted laboratory diagnostics [12,22].

In parallel, the convergence of digital pathology, automated cytology, advanced molecular diagnostics, and multi-omics technologies is expected to expand the diagnostic value of non-blood biological fluids. This convergence may enable more comprehensive disease characterisation and more precise integration of morphological, biochemical, molecular, and clinical signals. Its translation into routine practice will nevertheless depend on harmonised data models, multicentre data-sharing initiatives, and laboratory professionals trained in data quality, model validation, interpretability, and lifecycle monitoring [21].

Equally important will be the development of explainable AI models that provide transparent and clinically interpretable outputs. Explainability should be tailored to the needs of laboratory professionals and clinicians and should clarify the variables contributing to a prediction, the model’s uncertainty, and the circumstances in which expert review is required. Together with representative multicentre cohorts, prospective validation, and continuous post-deployment surveillance, these features may strengthen professional confidence, support regulatory evaluation, and improve generalisability across healthcare settings [23,24,25,26,27,28,37].

Ultimately, the success of AI in biological fluid diagnostics will not be determined solely by algorithmic performance. Its clinical value must be demonstrated through prospective implementation studies that assess analytical reliability, diagnostic performance, patient outcomes, workflow efficiency, cost-effectiveness, and equity. Only through the coordinated integration of technological innovation, analytical standardisation, interoperable data infrastructure, clinical validation, and expert human oversight can AI-driven CDSS become a reliable component of precision laboratory medicine. In this future model, AI should augment rather than replace the interpretative role of laboratory professionals [21].

## 9. Conclusions

The integration of artificial intelligence into the analysis of non-blood biological fluids represents more than a technological advance; it may contribute to a broader evolution in the role of laboratory medicine. As biological data become increasingly multidimensional, AI may support integrative models capable of combining biochemical, cytological, microbiological, molecular, imaging, and clinical information within a unified diagnostic framework. Its principal value therefore lies in supporting the analysis and interpretation of complex, heterogeneous data while preserving the central role of laboratory expertise and clinical context.

The current evidence base, however, remains heterogeneous and unevenly distributed across different biological fluids. AI-assisted interpretation of cerebrospinal fluid biomarkers and digital cytology of serous effusions currently represent the comparatively most advanced applications, whereas synovial fluid analysis and integrated multi-omics approaches remain predominantly at the proof-of-concept or early-validation stage. Even in the more developed fields, most studies are retrospective, use selected datasets, and lack sufficiently robust independent external or prospective validation. Nevertheless, the convergence of AI with high-throughput omics technologies may accelerate biomarker discovery, improve disease stratification, and create new opportunities for precision laboratory medicine.

Accordingly, AI systems should currently be regarded as tools that complement, rather than replace, laboratory expertise and established diagnostic pathways. Reported diagnostic performance alone is insufficient to demonstrate clinical readiness. Before routine implementation, AI-based Clinical Decision-Support Systems must demonstrate analytical robustness, reproducibility across populations and laboratory platforms, incremental clinical utility, and an acceptable impact on turnaround time, costs, and patient management. Methodological, analytical, regulatory, and ethical challenges must therefore be addressed before AI-driven tools can be implemented safely in clinical laboratories. Transparent reporting, clinically meaningful interpretability, human oversight, cybersecurity, and continuous monitoring for performance drift will be essential to maintain reliability throughout the model lifecycle.

Progress towards clinically useful AI will require investment in interoperable data infrastructures capable of connecting analytical instruments, middleware, Laboratory Information Systems, digital imaging platforms, and electronic health records. Harmonisation of specimen collection, pre-analytical handling, analytical procedures, data representation, matrix-specific reference intervals and clinical decision limits, and multi-omics workflows will be equally important. Representative multicentre datasets, independent external validation, prospective implementation studies, cybersecurity safeguards, and clear regulatory and governance frameworks will be required to ensure that AI models are transferable, safe, and clinically trustworthy.

Dedicated education and training for laboratory professionals will be another essential component of this process. Relevant competencies should include fundamental AI and data-science principles, assessment of data quality and representativeness, critical appraisal and local validation of predictive models, recognition of bias and dataset shift, interpretation of model outputs, and post-deployment performance monitoring. Laboratory professionals should remain directly involved in defining clinically relevant questions, evaluating analytical plausibility, supervising implementation, and determining when human review, recalibration, or model suspension is required.

Current evidence therefore does not demonstrate that laboratory medicine has already moved from individual biomarker measurement to the routine interpretation of complex biological systems. Rather, it suggests that AI may gradually facilitate this transition if supported by standardised laboratory processes, sustainable digital infrastructure, representative data, rigorous clinical validation, appropriately trained professionals, and continued expert oversight. Under these conditions, the integration of AI and multi-omics technologies may strengthen the contribution of laboratory medicine to precision healthcare while preserving professional judgement and patient safety.

## Figures and Tables

**Table 1:** Structured qualitative appraisal of representative AI studies in non-blood biological fluids. Study design, cohort representativeness, validation, performance, and risk-of-bias and applicability concerns are summarized. The appraisal was informed by PROBAST + AI [6] and TRIPOD + AI [7] principles; no formal overall risk-of-bias rating was assigned.

Biological Fluid	Clinical Question	Type of Data	AI Task	Representative Study	Study Design/Cohort Representativeness	Validation Strategy	Reported Performance	Risk-of-Bias and Applicability Concerns
Cerebrospinal fluid (CSF)	Data-driven profiling of Alzheimer’s disease and other neurological disorders	Core CSF biomarkers: Aβ42/Aβ40 ratio, total tau and phosphorylated tau	Unsupervised clustering and biomarker-based patient stratification	Bellomo et al. [14]	616 prospectively collected samples from two specialized Italian neurological centres	No independent external validation; clusters and cut-offs derived and evaluated within the study cohorts	Six biomarker-defined clusters and cluster-derived diagnostic cut-offs	Two specialized neurological centres and a heterogeneous control population limit cohort representativeness. The stability of the unsupervised clusters and derived cut-offs was not assessed in an independent cohort. Potential clinical impact and applicability to unselected routine populations were not evaluated.
Cerebrospinal fluid (CSF)	Alzheimer’s disease classification and proteomic biomarker discovery	CSF proteomic datasets from different cohorts and analytical platforms	SVM with recursive feature elimination and cross-validation (SVM-RFECV)	Hou et al. [26]	Retrospective secondary analysis of more than 1200 CSF samples obtained from multiple published datasets	Cross-dataset evaluation across ten cohorts and different analytical platforms; no prospective longitudinal validation	Accuracy exceeding 90% in several datasets	Secondary analysis of published datasets with incomplete demographic and clinical information; heterogeneous analytical platforms and potential batch effects; possible overlap among data sources not fully excluded; limited evaluation against other neurodegenerative diseases; no prospective assessment of calibration or clinical utility.
Cerebrospinal fluid (CSF)	Classification of normal cognition and different dementia stages	Conventional CSF biomarkers: Aβ1–42, total tau, phosphorylated tau and Aβ1–42/p-tau ratio	Supervised ML classification using boosted and bagged trees, logistic regression, SVM, KNN and Naive Bayes	Tiwari et al. [27]	Retrospective analysis of the NACC database; 696 patients in the MMSE-based analysis and 558 in the CDR-based analysis	Internal cross-validation only	Accuracy 84.4% for binary CDR-based classification and 75.4% for multiclass classification	Database-selected cohort not necessarily representative of consecutive patients; exclusion of records with missing data; class imbalance and a small severe-dementia subgroup; internal validation only; no independent assessment of calibration or clinical utility; results available as a preprint.
Serous effusions	Detection of metastatic breast carcinoma cells in pleural fluid	Cytological whole-slide images analysed through extracted image patches	Deep convolutional neural network classification	Park et al. [16]	Retrospective multicentre dataset of 569 pleural-fluid whole-slide images	Internal training, validation, and testing based primarily on image patches; no adequately powered independent WSI- or patient-level external validation.	Accuracy 81.1%; sensitivity 95.0%; specificity 98.6%	Patch-level rather than complete WSI- or patient-level diagnostic evaluation, with potential optimism if independence among patches from the same slide or patient was not fully ensured; selected quality-controlled images; manual annotations; binary classification without an atypical or indeterminate category; uncertain transferability to consecutive routine samples.
Serous effusions	WSI-level classification of benign versus malignant pleural effusions	Pleural-fluid whole-slide cytology images	Clustering-constrained attention multiple-instance learning (CLAM)	Kim et al. [28]	Retrospective nationwide dataset of 885 WSIs: 576 benign and 309 malignant	Internal model development and WSI-level evaluation; the additional evaluation dataset had limited negative representation; no adequately powered independent external validation.	Accuracy 97%; AUC 0.97	Retrospective quality-controlled dataset not clearly representative of consecutive routine samples; limited inclusion of atypical and technically suboptimal cases; markedly limited negative representation in the additional evaluation dataset; no prospective workflow or clinical-impact assessment.
Serous effusions	Detection and classification of mesothelial and lung-adenocarcinoma cells	Pleural-effusion cytological images with manually annotated individual cells	Object detection and instance segmentation using YOLOv8 and YOLOv11	Giarnieri et al. [29]	Single-centre dataset of 969 images containing 3130 mesothelial-cell and 3260 adenocarcinoma-cell annotations	Internal model evaluation only	Intersection over Union 0.72; YOLOv11 generally outperformed YOLOv8	Cell- and object-level rather than patient-level diagnostic evaluation; single-centre dataset; manual annotation; only two cell classes; no independent external validation or assessment on complete routine slides
Synovial fluid	Identification of monosodium urate and calcium pyrophosphate crystals	Raman spectra obtained from synovial-fluid samples	Principal component analysis followed by Support Vector Machine classification	Niessink et al. [30]	446 patients from three centres; 246 samples used for training and 200 for hold-out validation	Independent hold-out validation within the study; no external implementation cohort	Overall accuracy 88.0%; MSU accuracy 92.5%; CPP accuracy 96.0%	Dependence on specialized Raman instrumentation limits applicability to routine laboratories; limited evaluation of rarer crystal types and diagnostically challenging samples; only one ML approach tested; reproducibility across different instruments, acquisition protocols, and routine laboratory settings not established.

Note: Qualitative appraisal was informed by PROBAST + AI [6] domains (participants/data sources, predictors, outcome, and analysis) and TRIPOD + AI reporting recommendations. Attention focused on cohort and training-data representativeness, missing data, overfitting, validation, reporting, and routine applicability. Because reporting was incomplete and this was a narrative review, no item-level or overall risk-of-bias rating was assigned.

## Data Availability

No new data were created or analyzed in this study.

## Glossary

Artificial intelligence (AI)	Broad field involving computational systems designed to perform tasks that normally require human intelligence, such as pattern recognition, classification, prediction, and decision support.
Machine learning (ML)	Subfield of AI in which algorithms learn patterns from data to generate classifications or predictions without being explicitly programmed with fixed decision rules.
Deep learning (DL)	Subfield of ML based on multilayer artificial neural networks, particularly suited to analysing complex, high-dimensional data such as images, molecular profiles, and sequencing data.
Supervised learning	ML approach in which a model is trained using labelled data with known outcomes or diagnostic categories.
Unsupervised learning	ML approach used to identify patterns, clusters, or latent structures in data without predefined outcome labels.
Clinical decision-support system (CDSS)	Software designed to integrate laboratory, clinical, and other patient-related data to assist healthcare professionals in diagnostic or therapeutic decision-making.
Multi-omics	Integrated analysis of two or more molecular data layers, such as genomics, transcriptomics, proteomics, metabolomics, or metagenomics.
External validation	Evaluation of model performance using data that are independent of those used for model development, preferably obtained from different institutions, populations, time periods, or analytical platforms.
Explainable artificial intelligence (XAI)	Methods designed to clarify how an AI model generates its predictions and which variables contribute most strongly to individual outputs.
